# The evolution of language ideological debates about English and French in a multilingual humanitarian organisation

**DOI:** 10.1007/s10993-021-09586-0

**Published:** 2021-06-27

**Authors:** Maria Rosa Garrido

**Affiliations:** 1 University of Lausanne, Lausanne, Switzerland; 2 Bâtiment Anthropole, Université de Lausanne, section d’anglais, 1015 Lausanne, Switzerland

**Keywords:** Language debate, Humanitarian organisation, Linguistic requirements, Elite multilingualism, Mobile workers

## Abstract

This article traces the evolution of the ideological construction of elite multilingualism, with a focus on the values accorded to French and English, under transforming socioeconomic and institutional conditions at the International Committee of the Red Cross (ICRC). The ICRC, a major humanitarian agency based in Geneva, opens a window onto the construction of “internationalisation” and its accompanying language ideologies, resulting in fluctuating hiring requirements for “delegates” (expatriate representatives). The data include job advertisements for delegate posts from 1989 to 2020 complemented by interviews with different generations of delegates and ethnographic fieldwork in a recruitment fair. The analysis of language ideological debates at the ICRC illuminates the articulations and tensions between “roots” in Geneva, symbolised by French, and “routes” in its delegations worldwide, with English as a lingua franca, in dominant discourses about multilingualism. The requirements for ICRC delegates include English as a must and at least a second ICRC working language. Concerning the latter, there are tensions between the desired language regime at headquarters, privileging French as the “parent” language, and the current needs in key operations, with a shortage of Arabic speakers. The analysis shows that French requirements for generalist delegates have fluctuated from perfect command and good knowledge to an optional second working language. In the 2020 recruitment campaign, elite multilingualism is hierarchically stratified into English as a global language, other “working languages” including Arabic, and non-European languages such as Pashto or Dari as newly-introduced “assets”.

## Language policy and international expansion in a humanitarian agency

There has been a dramatic, uh, Anglosaxonisation of the ICRC [International Committee of the Red Cross], especially headquarters, over the past ten years. Amazing! We have transformed into, uh, we have transformed our i- identity of uh- Swiss-French uh- based uh, organisation, into a truly international one, a bit like the UN, where English is really lingua franca, and French is, definitely second, second one. [Interview with Gerard, 18-03-2016]

Gerard is a middle manager based at the International Committee of the Red Cross (ICRC henceforth) headquarters in Geneva. As we can see in the opening quotation, he links changes in language use in Geneva to socioeconomic transformations linked to becoming “truly international”. When he joined the ICRC at the turn of the century, he had to follow a three-week induction course in French despite his limited competences. In our interview, he claims that he was recruited because there were not enough candidates and because of his fluency in another working language needed in operations. He recalls that meetings in Geneva were “still in French” in 2007 whereas “now they’re all English”. Above, he makes a connection between the increased importance of English as lingua franca, to the detriment of French as a parent language at headquarters, and the institutional transformation of the ICRC from a Swiss organisation to an international one. This transformation was initiated after the opening of “delegate” (expat representative) posts to non-Swiss nationals in December 1992, informally known as “internationalisation” among delegates. The ICRC is caught in the tensions surrounding English as a dominant lingua franca vis-à-vis the traditional parent language in the organisation’s headquarters, documented in multinationals (Lønsmann, 2014). Many international organisations grapple with a practical dominance of English despite having a multilingual policy for internal documents and meetings (de Varennes, 2012). Even if languages are designated as “official” or “working”, this does not mean that they are equal to others of the same rank, with an increasing trend towards English-only in internal meetings.

The ICRC’s shift from a Swiss-French identity, represented by Swiss delegates and its Genevese roots, towards a global workforce that privileges mobility for international delegates has resulted into on-going debates about the dominance of English over French and the perceived “Anglosaxonisation” of management strategies. The latter are said to be typical of multinationals and UN bureaucracy among delegates (Bussard, 2018, see Gerard’s quote above). The previous Director-General, Yves Daccord, claimed that “with an organisation that is growing, we increasingly work in English, but I want to keep French because in terms of mindset and culture, we have to stay tuned to different frequencies” (Benoit-Godet & Bussard, 2018, my translation). Kim and Schneider (2008) argue that “French remains important to the identity of the ICRC as it differentiates it from other UN and humanitarian agencies” (p.7). These language debates reflect the tensions between roots and routes, that is, between place attachment and mobility, namely between a history of Swiss Francophone roots, partly through preserving French as a mandatory language for delegates, and future routes, seeking to “transcend its Western image and move towards an even more global culture” (Brühwiler et al. 2019: 14). Routes and roots of course coexist, as “human location is constituted by displacement as much as stasis” (Clifford, 1997: 2), and are combined to create a system of differentiation between languages and speakers/workers in this organisation.

To date, there are no studies of internal language policy in international humanitarian organisations whose mission and structure differ from those of multinationals and multilateral agencies. In addition to contributing to a broader understanding of language policy in a lesser-studied type of institution, this article engages in an interdisciplinary dialogue with the fields of international relations and development studies. One of the main debates centres on the localisation of humanitarian projects and (Western) international organisations. Thus, this process emphasises enhanced collaborations with local partner associations and improved communication with beneficiaries, but pays little attention to language in institutional policies (Footitt, Crack and Tesseur, 2018). This article will historically trace the continuities and ruptures in the ideological construction of elite multilingualism (Barakos & Selleck, 2019) for access to prestigious delegate positions, with a focus on French and English, under transforming socioeconomic and institutional conditions at the ICRC. The present study is based on a novel corpus of job advertisements for ICRC delegates since 1989–2020, complemented by interviews with different generations of delegates and ethnographic observations in a recruitment fair.

After this introduction, the Sect. “Roots and routes: An international organisation in Geneva” will historically and politically situate the ICRC’s origins in “International Geneva” and connect its international expansion with its changing language policy and vocabulary. The Sect. “Language ideological debates and the construction of multi-layered elite multilingualism” will conceptually articulate the evolving language ideological debates at this institution with the construction of a multi-layered, fluctuating elite multilingualism for expatriate humanitarian posts. The Sect. “A critical discursive approach to language policy” will then outline the critical, discursive and ethnographic approach to language policy adopted to make sense of the historical evolution of language ideological debates and policies at the ICRC. The next section, “Multilingualism and “internationalisation”: Continuities and ruptures in language debates and policies”, will trace the connections between “internationalisation” processes and the broadening of elite multilingualism with a focus on the institutional role of French vis-à-vis English over the span of three decades. The last section will conclude that English-centric multilingualism has gradually oriented towards “internationalist” narratives favouring humanitarian routes, with a broadening of strategic languages, in tension with “traditionalist” narratives of Genevese authenticity symbolised by French, whose ambivalent value over time indexes this on-going debate.

## Roots and routes: an international organisation in Geneva

The International Committee of the Red Cross (ICRC) is a humanitarian organisation with roots in Geneva and routes spanning to over 80 countries. It is legally constituted as an association under Swiss law with an international mandate based on the Geneva Conventions since 1864. Its mission includes protection activities (such as confidential visits to detainees), assistance to the affected populations (such as healthcare) and prevention (mainly through the dissemination of International Humanitarian Law) in emergencies and armed conflicts. It is primarily funded by voluntary contributions from wealthy signatories to the Geneva Conventions (around 80–85% of its budget), especially the United States and the Swiss Confederation (Forsythe, 2005: 233). Its major operations in 2017 included Syria, South Sudan, Iraq, Yemen, Somalia, Afghanistan and Nigeria, with the first five in Arabic-speaking designated regions. Today, the ICRC has English and French as “administrative languages”, with less widely used “working languages” for operations, including “regional languages with an international dimension (Arabic, Spanish, Portuguese) and national languages with a regional or sub-regional dimension (Russian, Chinese)” (Krimitsas, 2012: 2–3).

The ICRC is historically rooted in Geneva and “neutral” Switzerland. Unlike later international organisations that set their headquarters in Geneva, notably the United Nations (UN), the ICRC was founded in Geneva in 1863 by the local Protestant upper classes following Henri Dunant’s initiative to assist wounded soldiers after witnessing the Solferino battle (see Forsythe, 2005). At present, the name of the city features in the ICRC logo that reads “Comité International Genève” (in French) circling a red cross against a white background. The history of humanitarianism in Geneva started with Italian and especially French religious refugees fleeing to this Protestant Calvinist centre from the sixteenth century onwards, which became “a bastion of the Huguenot international” (Kuntz, 2010: 16). In the nineteenth century, the foundation of the ICRC (1863), as well as the first Geneva Conventions (1864), enhanced Geneva’s positioning as a centre for international cooperation.

Swiss political neutrality (1815) and multilingualism are central pillars in the construction of “International Geneva” as a humanitarian hub. At a federal level, Switzerland has been constructed as “an exception” to homogenising, monolingual European nation-states because it is presented as a nation created out of the will of different linguistic and cultural groups to live together. This argument was used in 1919 by Lord Robert Cecil, leading architect of the League of Nations, to support Geneva for its headquarters as proof of Swiss “absolute neutrality”. Despite federal multilingualism, the vast majority of cantons and municipalities are officially monolingual. The city of Geneva is officially Francophone while it is home to people from all over the world, many working for international organisations. There are tensions between the city’s Francophone identity, anchored in its Protestant history and roots, and the emerging multilingualism, connected with international organisations and their mobile employees.

Swiss neutrality and humanitarian neutrality reinforced each other for decades. The original Red Cross emblem has inverted the colours and retains the shape of the Swiss flag. However, the historically close relations with the Swiss Confederation were diluted after the headquarters agreement in 1993. Separating humanitarian neutrality from Swiss political neutrality had become necessary due to Switzerland’s referendum on EU membership (December 1992), which obtained a negative vote, and Switzerland’s later entry into the United Nations in 2002 (Julier, 2002; Troyon & Palmieri, 2007). Today, it is the Swiss Confederation that uses the Red Cross for nation branding rather than the ICRC using *Swissness* to promote its efforts (Brühwiler et al. 2019). According to former Director-General Yves Daccord, it is important that interlocutors perceive the ICRC not as Swiss but “as a humanitarian organisation based in Geneva”, a city which has a particular status in the world (Benoit-Godet & Bussard, 2018, my translation). This echoes the perception of the UNHCR headquarters in Geneva as “international ‘headquarters’ and not as a place geographically located on Swiss territory” (Fresia, 2009: 180). Therefore, the ICRC’s roots have been detached from the nation-state, Switzerland, and identified with a city historically crisscrossed by international routes, as we saw earlier.

A major process that decoupled the ICRC from the Swiss Confederation was the opening of “delegate” positions to international candidates in 1992, which had been reserved for Swiss nationals to ensure their neutrality during the Cold War period (Palmieri, 2012: 1286).^1^ This decision was due to the opening of new humanitarian routes. Following an upward trend since the 1970s, the 1990s saw an increase in humanitarian donations following the mediatised crises in Iraq, Bosnia-Hercegovina and the African Great Lakes region (Carbonnier, 2015). In 1991, the ICRC exponentially grew in terms of personnel working in the field and its budget, + 161% than the previous year (Palmieri, 2012: 1294). Owing to the saturation of the Swiss labour market for generalist and specialist profiles (Julier, 2002: 36), the ICRC had to tap onto a pool of international candidates to represent this Genevese institution. In 2016, the ICRC employed 15,000 workers across the globe and only 2,127 (around 14%) were “mobile staff” (ICRC webpage, 2016), a larger category of expatriate employees including generalist “delegates” that move between temporary missions in the network of over 80 delegations worldwide. They are more visible, have more authority and enjoy better conditions than resident (or “national”) staff based in a national delegation. “Delegates” have traditionally formed the ranks of future managers in Geneva, as field experience and mobility are greatly valued in the humanitarian sector (Kim & Schneider, 2008: 11). Many of them become (sub-)heads of delegations and eventually managers at headquarters. According to Kim and Schneider (2008), 47% of expats and 80% of managers had Swiss nationality in 2006.

As a result of this geographical and personnel expansion, the ICRC has become a “humanitarian enterprise” with a vocabulary embedded in marketing, economics and trade and an emphasis on accountability to donors through quantifiable results (Palmieri, 2012: 1294). The tensions between routes and roots were further accentuated.

by its desire to be present globally while keeping its main decision-making centre in Geneva, but also through the relocation of some of its services abroad for financial reasons, the ICRC corresponds, in a sense, to the common definition of the multinational, although, again, its fundamental objective differs from that of multinational firms. (Palmieri, 2012: 1295)

The on-going decentralisation of certain services such as IT, translation and communication has been criticised in Genevese newspapers and by some Swiss “old-timers” because, to them, the ICRC is becoming like any other UN agency to the detriment of its origins. In 2013, the then Director-General Yves Daccord emphasised that the ICRC is “staying true to its roots” in Geneva while asserting “the need to adapt to a changing world” in terms of further internationalising its workforce, diversifying funding sources and adapting to ICT (ICRC webpage). Concerning the international workforce, he later declared that “a Swiss candidate does not always fit the bill” in global recruitment campaigns in which their “main problem concerns languages”, with only 12% of staff speaking English and French (Benoit-Godet & Bussard, 2018, my translation). In the next section, I will articulate these socio-politically situated debates about language at the ICRC with the construction of a multi-layered multilingualism, simultaneously orienting to Genevese roots and international routes, for humanitarian employment.

## Language ideological debates and the construction of multi-layered elite multilingualism

In order to grasp the tensions between roots and routes at the ICRC, this article will explore the *language ideological debates* about what language varieties count and their institutional status and value, based on their *linguistic authority* as anonymous and/or authentic. As a result of these language ideological debates, a multi-layered and fluctuating *elite multilingualism* allows access to prestigious and coveted “delegate” posts. Named languages are given a different value according to the *scale* that they are imagined to index on a continuum from local to global, with institutional strategic multilingualism orienting to multiple centres of linguistic authority.

The analysis will trace the evolution of *language ideological debates* that articulate, produce, change and enforce certain language ideologies within a wider socio-political and historical background of power relations, discrimination forms and identity construction (Blommaert, 2001). Language ideologies are not *only* about language. They are “the cultural system of ideas about social and linguistic relationships, together with their loading of moral and political interests” in a cultural setting (Irvine, 1989: 255). Language use is understood as indexical of people’s character, social class, etc. and this impacts social judgements, especially in recruitment contexts (Roberts, 2013). Language ideologies also give value to certain languages and their speakers in multilingual contexts. Heller (2007:5) defines them as “discourses in which processes of attribution of value to linguistic forms and practices are inscribed, along with the processes of construction of social difference and social inequality within which they are associated”. To understand the values accorded to different language varieties in these debates, I will draw on the two bases of linguistic authority, *authenticity* and *anonymity,* proposed by Gal and Woolard (2001). Authenticity regards language as an ethnic marker “from somewhere”, grounded in a territory, whereas the ideology of anonymity constructs a public, standard and universal voice “from nowhere”. Anonymity refers to the unmarked language that belongs to no-one and is thus imagined to be universally accessible to all, useful for “routes” across territories. Authenticity, by contrast, regards the primary language as the genuine expression of an imagined community or a person’s essential self, linked to historical roots.

These language ideologies define a certain form of multilingualism required from this elite minority of humanitarians that is suitable for routes (field delegations), through universally-accessible lingua francas like English, and roots (headquarters), indexed by French as the authentic parent language. These language ideologies link certain languages with an idealised “delegate” persona ready to navigate routes and represent roots. Language ideological debates define who gets access to this coveted post through a required form of *elite multilingualism*:

a phenomenon that brings social and/or material capital, a sense of belonging, prestige, excellence, privilege, and access through the use of specific linguistic resources for certain social groups and individuals. Elite multilingualism is essentially a phenomenon where language serves as an access code to a local, national or global perceived elite (way of life). (Barakos & Selleck, 2019: 362)

Therefore, it is an inherently ideological construction for gatekeeping. Language ideological debates at the ICRC centre on what counts as “elite multilingualism”, i.e. which “languages” are of strategic value to the institution, which institutional status they occupy in a multi-layered hierarchy (“administrative”, “working” etc.), and which ones are required for “delegate” posts in a changing institutional and socioeconomic landscape. This construction of multilingualism for humanitarian work forms the basis for language policy entextualised in artefacts like recruitment campaigns with linguistic requirements and “assets”. Put differently, elite multilingualism mediates the relationship between the institutional value of “languages”, as named objects that are tested and certified as technical skills, and access to employment as an ICRC “delegate” and its promise of career advancement. The definition and measurement of elite multilingualism, typically including two or more internationally useful languages, becomes a terrain for exclusion and distinction. Elite multilingualism is thus multi-layered, as it is rather hierarchical and ideologically loaded in a given context.

The concept of *scaling* (Blommaert, 2007a) opens a window onto the complex layers and nuances of linguistic resources in this elite multilingualism for delegates. Scaling is a process of hierarchical ordering of different linguistic resources according to the scale they are ideologically imagined to operate in, namely, local, national or global along a continuum with intermediary scales (Blommaert, 2007a). It establishes a metaphorical relationship between social hierarchisation, sociolinguistic processes and their distribution in space. Elite multilingualism may encompass different linguistic resources along this local–global continuum. Scale-making not only compares several “languages” but also allows to determine their relative value. In other words, not all the language varieties are valued equally in a certain construction of elite multilingualism, signalled by language ideological debates about their institutional status. The arguments for their institutional value are based on connections to local/ national roots, linked to authenticity ideologies (voices “from somewhere”), and global/ regional routes mainly indexed by “anonymous” languages for broader communication. It is important to note that global scales are not intrinsically superior to other scales because local scales might also be prestigious alongside global ones (Prego Vázquez, 2020), as we saw with Geneva as a Francophone city and international centre in the previous section.

“Jumping” these scales depends on speakers’ unequal access to discursive and linguistic resources that index and iconise certain scales. This evaluative authority emanates from “multiple real or perceived centres” in a continuum from local to global to which speakers and institutions orient in their interactions (Blommaert, 2007b). In communicative practices and language debates, the different socio-spatial centres are simultaneously layered, articulated and projected. As a comparative and evaluative endeavour, scaling might connect and even conflate what is geographically, geopolitically, temporally or morally “near” in opposition to what is “far” (Carr & Lempert, 2016:3). Scale shifts trigger changes in value for language varieties and they are negotiated in social life, as people and institutions conceive, cultivate, put to practice and shift scales (Carr & Lempert, 2016). The institutionalisation of elite multilingualism involves the evaluation of cases against rules and regulations (Blommaert, 2007a) and the privileging of certain voices and positions at the expense of others (Carr & Lempert, 2016) in the access to “a global perceived elite” of humanitarians.

English, in particular, is semiotised as being the emblem for international mobility, or routes, to improve one’s socioeconomic chances (Blommaert, 2007a: 13) in multilingual policies and repertoires. As a product of scaling, globalised English is often conceived of as generalisable to all spaces and speakers in contrast with other languages and varieties bound to specific spacetimes as “authentic” indexes, offering less translocal mobility (Blommaert, 2007a). English is a prestigious lingua franca connected with “expats” working for international organisations in Geneva and in field delegations. The dominant role of English is generally presupposed in international organisations, often replacing French as the anonymous lingua franca in diplomatic settings (Wodak, Krzyzanowski and Forchtner 2012: 167). Lønsmann’s study of a multi-national in Denmark (2014) documents the struggle between different ideologies of nation-building and internationalisation, indexed by Danish as the authentic “parent company language” and anonymous English as a “corporate language” respectively, in language ideological debates. She concludes that English and the headquarters language are both needed for full participation and career advancement. The drive towards English is often pitted against a concern and a need for maintaining institutional language diversity. For example, multilateral organisations like the EU tend to favour a set of working languages (Wodak et al. 2012) whereas “strategic multilingualism” in international NGOs like Amnesty International (Tesseur, 2014) is designed to increase the organisation’s impact and growth in adapting to the changing structure. The top-down definition of language resources as potentially “strategic” is based on re-orientations to centres of linguistic authority associated with operational needs and recruitment pools.

As a result of on-going language ideological debates, fluctuating constructions of elite multilingualism have granted access to elite expatriate positions at the ICRC over time. In the next section, I will outline the critical discursive and ethnographic approach to language policy as a multi-layered phenomenon anchored in specific sociopolitical and institutional contexts.

## A critical discursive and ethnographic approach to language policy

To analyse scaling processes in language debates about “strategic” multilingualism at the ICRC, I will combine a discursive, practice-based approach to language policy within institutions with a critical, ethnographic lens accounting for power dynamics and material consequences.

I draw on a *discursive approach* to language policy as “a multi-layered phenomenon that is constituted and enacted in and through discourse” (Barakos & Unger, 2016:1). Language policy is conceptualised as a dynamic and complex process involving institutional policies, resulting from language debates and entextualised in artefacts, and on-going interpretation and appropriation in the actual practices of social actors over time (Johnson, 2009). A practice-based approach to language policy moves “beyond the text” in ways that engage with discursive spaces, policy actors and individual experiences anchored in specific socio-political and historical contexts (Barakos & Unger, 2016). Concerning institutional settings, Duchêne (2008) defines “discourse as the place of emergence, crystallization and materialization of the positioning of actors and institutions” (p. 30). Discursive data need to be considered in terms of their historical emergence in the course of the organisation’s history, how they are transformed over time and how certain historical developments influence them (Duchêne, 2008: 38). In a historiographic fashion, I will articulate major institutional developments discursively linked to “internationalisation” projects with language ideological debates and multilingual requirements over the span of three decades.

In line with Duchêne’s definition of discourse as the locus of emergence and transformation of an institution’s positioning over time, Tollefson’s *critical approach* to language policy (1991) maintains that language policies serve or undermine given socio-political, economic and institutional interests. This approach is thus concerned with language ideologies that institutionalise certain language varieties with material and symbolic consequences for social actors. Blommaert (2001) calls for a “historiography of language ideologies” (p.1) in specific contexts to investigate political interventions, agency and power with “an ethnographic eye” (p. 7) on the discourse producers and institutional actors, their interests and their alliances. Therefore, a historiographic approach to institutional language policy must be combined with an ethnographic approach to practices and actors “beyond the text”. As a critical approach, the ethnography of language policy regards language and communication as a terrain for struggles about power relations and access to other symbolic and material resources. It

[…] can include textual and historical analyses of policy texts but must be based in an ethnographic understanding of some local context. The texts are nothing without the human agents who act as interpretive conduits between the language policy levels. (Hornberger and Johnson 2007: 528)

Heeding Barakos and Unger’s practice-based approach, ethnographic fieldwork also allows us to provide an account of the institutional trajectories and networks through which these discourses and documents circulate and are reproduced, negotiated and resisted by various policy actors.

In keeping with this critical discursive and ethnographic approach to language policy, my analysis is based on the triangulation of institutional texts and ethnographic data. I initially became familiar with the tensions between English and French at the ICRC headquarters thanks to 9 interviews with established Swiss delegates—including Gerard—and later on, 11 interviews and 3 focus groups with newer recruits working in the Middle East and Northern Africa (MENA henceforth). In 2016, I observed a recruitment fair in Lausanne targeting Swiss university graduates. All the informants’ names in this article are pseudonyms and their identities have been anonymised to the greatest extent possible. In parallel, I have compiled a corpus of job advertisements and recruitment materials (1989–2020) from the ICRC library and webpage.

In the analysis below, I will weave these historiographic and ethnographic data to trace the evolution of language ideological debates since the 1992 opening of “delegate” positions to non-Swiss nationals. Through a critical lens, the fluctuating construction of strategic multilingualism over time will be linked to language debates and policies in an institutional context marked by “internationalisation”.

## Multilingualism and “internationalisation”: continuities and ruptures in language debates and policies

Based on major institutional developments in the HR management of delegates, I have traced the continuities and ruptures in language ideological debates on the role of French in relation to English and in the construction of elite multilingualism for delegates. First, I will link the gradual opening of “delegate” posts to non-Swiss candidates in the 1990s—known as “internationalisation” among delegates and managers until today—with the continued hiring requirements of French and English and an increasing interest in other “working languages” for new routes. Second, I will show that the increasing number of international delegates at the turn of the century sparked a debate about “Anglosaxonisation” as a threat to linguistic diversity and French in Geneva, revealing tensions between routes and roots. Meanwhile, academics, HR and some delegates were concerned with the ICRC delegate’s “Western” profile and called for more heterogeneous profiles, especially Arabic speakers for new operations. Last, I will scrutinise the multilingual turn embedded in the single global workforce framework, which I understand as the latest stage of “internationalisation”. Since 2016, recruitment campaigns construct an English-centric, hierarchical model of multilingualism including more non-European languages as “assets” for routes and relaxing French requirements in a gradual detachment from the roots, the latter engendering criticism in Geneva.

### French–English bilingualism for non-Swiss delegates (1990s)

**Figure 1:**
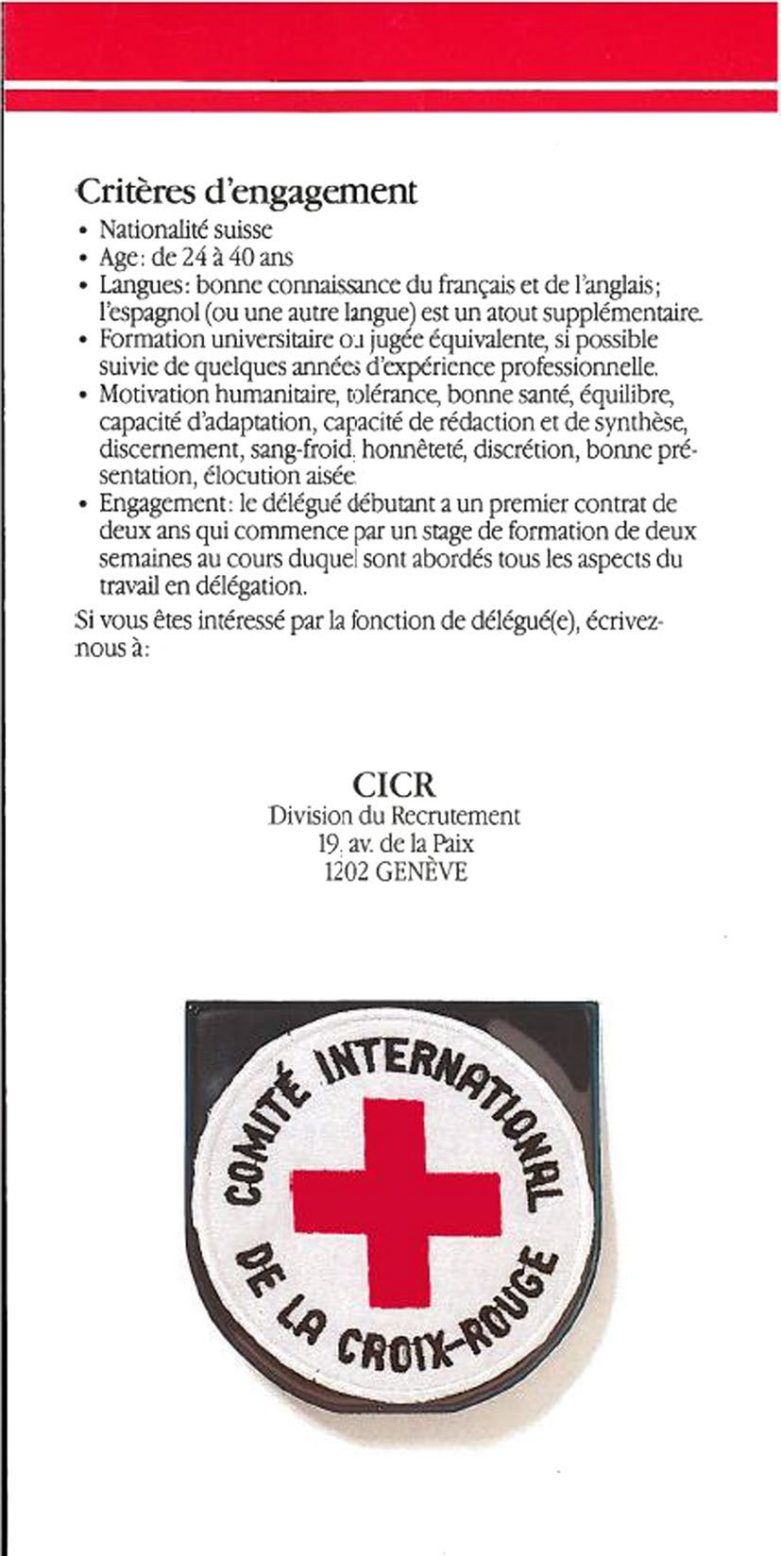
Leaflet. *Profession: Délégué(e*), 1st edition, 1989 (*Source*: ICRC library, permission for reproduction given by email)

Let us go back to the institutional roots. Before 1992, ICRC delegates were recruited from a Swiss national pool of university graduates for a generalist profile. In 1989, a recruitment leaflet for “delegates” (see Figure 1) presents the ICRC as a “Swiss, independent humanitarian institution” at a national scale. Accordingly, it was only published in the two main “national” languages, French (official in the Geneva canton) and German (the majority language in Switzerland). Among the criteria for employment, we find Swiss nationality first and “good knowledge of French and English, Spanish (or another language) being an added advantage” (see Figure 1). Please note that the bilingual requirement puts French before English, privileging the national/local scale. The construction of elite multilingualism was based on French as the internal working language and English as an international lingua franca in the field, with Spanish as an asset for operations in Central and South America. In Figure 1, Spanish is singled out in a vague definition of multilingualism (“or another language”) that does not list other useful languages. This might be due to the restricted pool for candidates in Switzerland: multilingual demands could have further limited the number of eligible candidates at a national scale. This limited pool could explain why “some years of professional experience” is presented as desirable (“if possible”) rather than a requirement. Besides, German was not required for employment but it was used to advertise positions.

**Figure 2:**
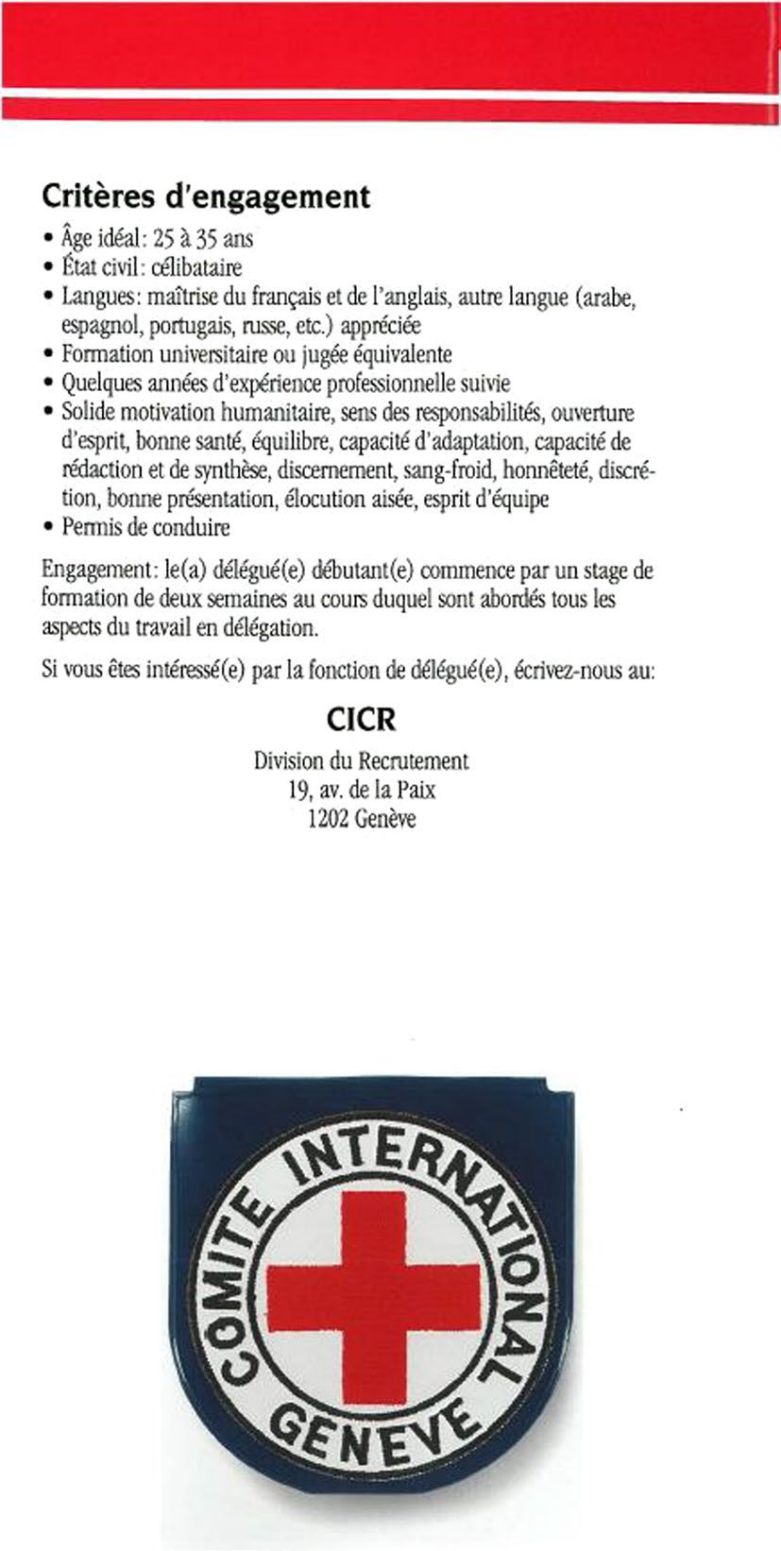
Leaflet. *Profession: Délégué(e*), 3rd edition, 1997 (*Source*: ICRC library, permission for reproduction given by email)

The financial, geographical and personnel expansion of the humanitarian sector motivated the opening of delegate positions to non-Swiss nationals (see Contextualisation). This marks the onset of tensions between roots and routes in institutional debates and decisions over the role of French. The leaflet above (Figure 1) was revised and used for later recruitment campaigns, of which the third edition published in 1997 is preserved at the ICRC library (Figure 2). It was re-edited in the same two languages at a time when Swiss delegates were still the majority. In Figure 2, the ICRC presents itself as an “independent humanitarian institution” without the earlier reference to Switzerland because the institution jumped scales from national to international. As we saw earlier, the ICRC logo contains a reference to Geneva in French, which indexes the continued importance of Geneva as a centre of institutional and linguistic authority. The nationality restriction is gone in this edition but the new requirements, linguistic and otherwise, are much more demanding to cater for new routes. Apart from being single and having a driving permit, the desired delegate profile now requires professional experience. The job advertisement reads: “Command of French and English, another language (Arabic, Spanish, Portuguese, Russian, etc.) appreciated” in an incipient hierarchical construction of elite multilingualism. Towards the end of the decade, the two administrative languages, presented in the same order, remained compulsory in order to recruit Swiss-like profiles suitable for both roots and routes, but the ICRC required better competences (from “good knowledge” to “command”). In this leaflet, we have a broadening of elite multilingualism to a list of major anonymous lingua francas as a bottom layer of desirable strategic multilingualism for regional operations as a new scale, which are today’s “working languages”.

The main differences between these two leaflets show the transition from a Swiss to an international pool of recruitment of candidates with a similar profile to that required in 1989. In 1993, the recruitment division established its official position vis-à-vis the opening of “delegate” posts to non-Swiss delegates: it would primarily recruit Red Cross/Red Crescent National Society collaborators with over 36 months of field experience and who conformed to shared values. However, direct recruitment of non-Swiss delegates without ICRC field experience took off in 1995 out of pragmatic needs (Julier, 2002: 43) because they did not have enough candidates, as this 1997 leaflet proves. In 1999, the Assembly proposed a ratio of a 1/3 of non-Swiss delegates (Julier, 2002: 43). This can be interpreted as a conflation of what is morally, linguistically, and geopolitically “near” (Carr & Lempert, 2016:3) to the ICRC Swiss delegates, maintaining French as the authentic parent language, in this unprecedent scale jump to an international market. In practice, this form of multilingualism not only created some continuity but also practically reduced this jump to a regional (European) scale (see next section). The main ideological change is the scale shift from Swiss nationality as a guarantee of neutrality to a gradual opening to “acceptable” nationalities in the contexts where the ICRC operates (Julier, 2002: 42). The institution made a considerable effort during initial training to create an “ICRC nationality” equivalent to “neutrality, independence and confidentiality” (Julier, 2002) and there was even discussion of developing an ICRC “passport” (Forsythe, 2005: 232), moving away from national constructions of Swissness to an emerging post-national category. In fact, this was not successful and nationality remained important in the recruitment of “delegates” (Garrido, 2021).

In addition to debates on nationality, which language ideological debates emerged in this period of “internationalisation” understood as the opening to a global labour market? Paul, a Swiss Francophone who was a delegate from 1979 to 2010, recalled that language competences other than basic skills in English were not required when he was recruited (see Excerpt 1 below). He termed it a “second language” (lines 8–9) without specifying a first language at the ICRC, thus taking French as the (authentic) ICRC language for granted. When I asked about French (line 10), he explained that it was required or highly recommended for those who wanted to eventually work at headquarters (lines 11–12), where French was primarily used and he claimed that it is “logical” to be able to speak French in Geneva (line 21). Paul clarified that the ICRC delegates concerned would be Swiss Germans and English speakers during that period (lines 17–19), which shows a mix of Swiss and international candidates. Paul stated that English was gradually used as a working language in Geneva throughout the 1990s (lines 24–25), a decade earlier than in Gerard’s account. Therefore, he constructed a dichotomy between French as the local language “from somewhere” i.e. Geneva, highly recommended for career advancement, and English as an international language “from nowhere”, a must for missions since the early days of the recruitment division. Paul constructed two coexisting centres of linguistic authority, the taken-for-granted local (Geneva) linked to authenticity and the international (field missions) indexed by anonymous English, without any mention of other valuable languages.

Excerpt 1. Interview with Paul, retired delegate. 03-02-2016.
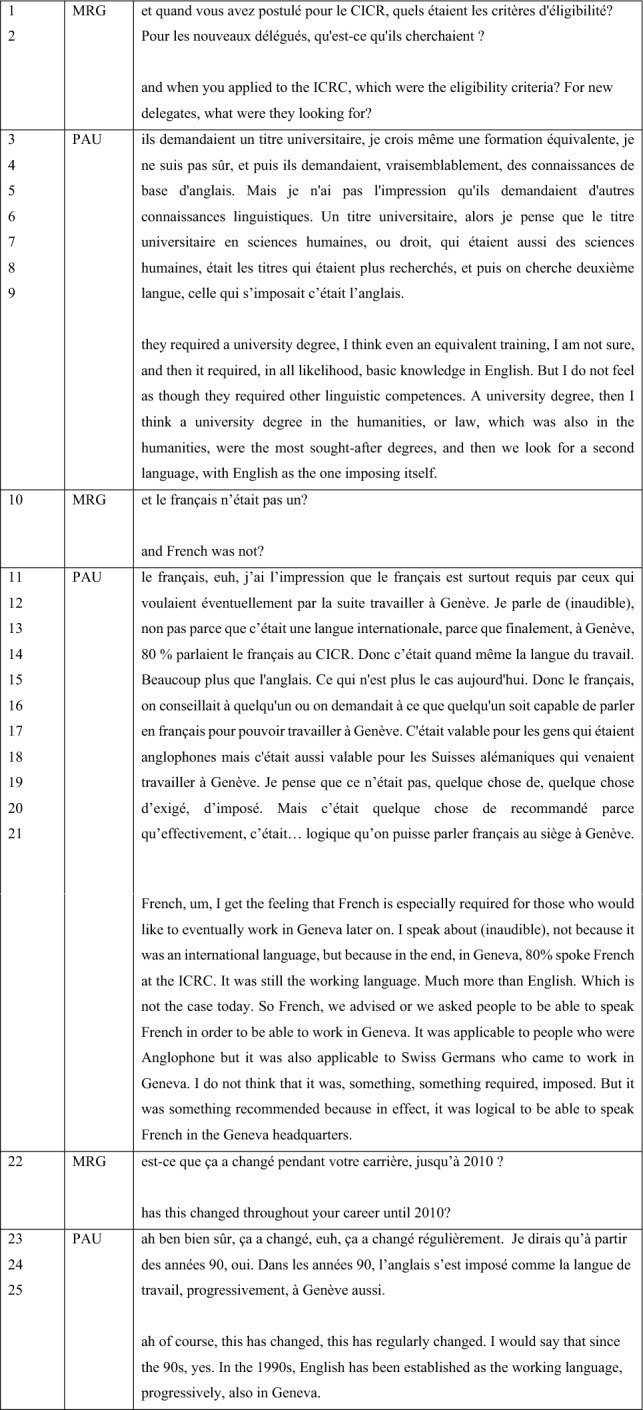


Despite the increasing use of English at headquarters (see Excerpt 1), the gradual opening to non-Swiss nationals and the dominance of Swiss delegates translated into a maintenance of French as an internal language for training and recruitment in the 1990s and beyond, according to Gerard. For example, Carolyn, a Swiss German who was recruited as a delegate in the early 1990s, moved to Lausanne to improve her French before the ICRC induction course at her own initiative.

In the 1990s, elite multilingualism at the ICRC centred on English- French bilingualism with some international languages, notably Spanish, as “assets” for humanitarian routes. The gradual opening of “delegate” posts involved a gradual shift from the national (Swiss) scale towards a European scale owing to French requirements and “acceptable” nationalities for neutral representation. This process triggered institutional debates about the ICRC’s Francophone roots in this expansion for new routes, as we shall see next.

### Ambiguous role of French and discourses of “Anglosaxonisation” (2000–2015)

The “internationalisation” process at the ICRC, initially understood as the gradual opening to non-Swiss delegates in the 1990s, was depicted to have mainly benefitted “Westerners” (left undefined) because HR targeted Western pools and institutions (Julier, 2002: 6). For many HR informants at the time, the opportunity to “dewesternise” the ICRC was “wishful thinking” in a global international organisation with most field operations outside Europe (Julier, 2002). Troyon and Palmieri (2007) claim that the “delegate” profession had been “at least Westernised” (p.110): 65% of “non-Swiss Westerners” were recruited in 2006, compared to only 11% of “non-Westerners”. They do not define either category. In 2001, 40% of delegates were non-Swiss because of the difficulties in recruitment and despite the 1/3 quota in place (vs. 2/3 of Swiss candidates). In the face of this incipient diversification of mobile staff, the fear of “Anglosaxonisation” was common among the Swiss delegates that Julier interviewed (see Excerpt 2).

Excerpt 2. Interview with Swiss delegate. Source: Julier (2002: 104).

the maintenance of French, alongside English, clearly inscribes itself in the heritage and the cultural identity of the ICRC, which has its roots and its headquarters in a Francophone city. The adoption of English as the only working language would not be a cultural enrichment (my translation).

This delegate, like the Director-General Daccord above, regards French as an authentic language representing the cultural identity of this institution in Geneva, its roots. In this discourse, institutional policies favouring one major lingua franca, in this case English, are opposed to the defence of cultural and linguistic diversity, encompassing the preservation of the city’s and the institutional Francophone identity. This discourse of diversity is mobilised by *la Francophonie* against the perceived Anglo-American imperialism and linguistic homogenisation of the world (Vigouroux, 2013).

As the “internationalisation” process was gradually eroding the dominant position of French at headquarters, the language ideological debate about the institutional status of French as the “parent language” intensified. In 1998, the ICRC Assembly decided not to designate English and French as “official” languages above other working languages and there was no general language policy at the turn of the century (Julier, 2002:107–110). Julier (2002) highlights the ambiguity of the status of French, as either a requirement on par with English or as an added value. For example, English language courses were subsidised by the ICRC while French language courses were not. The eventual designation of French and English as administrative languages in 2003 sought to promote higher levels of (passive) French competences among employees. A 2004 recruitment brochure for delegates, communication delegates and interpreters/translators was published in French and English as institutional languages, rather than German and French as Swiss national languages. The hiring requirements included “excellent command of English (and good command of French for delegates)” which actually gave more weight to English competences than to those in French. French was only required of delegates, who traditionally formed the ranks of future managers in Geneva and in delegations (Kim & Schneider, 2008:11). Other languages were not mentioned in this general brochure for different types of language workers.

By contrast, the 2012 booklet “Working for the ICRC” was published only in English. To become a delegate, the requirements included “excellent command of English, good grasp of French and other useful languages indicated on the ICRC’s website”. Although “good” French was still required, it was second to full command in English (unlike earlier campaigns in the previous section). Multilingualism (“other useful languages”) was not defined and was probably restricted to the list of major world languages on the 1997 brochure (Arabic, Spanish, Portuguese, Russian etc.). Thus, the desired “delegate” in the early 2000s had a similar linguistic profile to that of the nineties but French requirements were actually relaxed, contrary to the 2003 institutional decision to strengthen its role. This leaflet explained that interpreters/translators use English as a pivotal language. Communication delegates were subject to the same linguistic requirements as generalist delegates because they had to carry out one mission as a generalist delegate before joining the specialised Communications pool. In 2012, this specialised pool targeted a language-specific Arabic profile owing to the shortage of Arabic speakers for the on-going MENA operations (see Excerpt 3). In this case, French is not required for employment but it is connected with career progression as an administrative language linked to headquarters in Francophone Switzerland. Arabic is useful with interlocutors in MENA but it does not constitute an asset for management positions and missions outside the region (Hassemer & Garrido, 2020).

Excerpt 3. Job advertisement for “Arabic-speaking communication delegate” (2012), sent by one of my informants.

Excellent command of Arabic and English. In addition, a good command of French is a distinct asset for career development within the ICRC, as French is an institutional language.

Overall, the recruitment campaigns analysed in this section suggest a relaxation of French competences for delegates despite the 2003 policy that makes it equivalent to English as an administrative language. This was partly due to the difficulties in recruiting multilingual delegates with French competences in an expanding international pool of candidates, especially those who speak “strategic” languages needed in the field. Prior to 2012, French was a deal breaker for qualified Arabic-speaking communicators. Alex was one of the delegates who replied to the job advertisement for Arabic-speaking communication delegates (Excerpt 3 above). In his early thirties, Alex held a BA and MA from the American University of Beirut. Upon graduation in 2006, he wanted to apply to the ICRC but he was officially told that he needed to speak French. He was “pissed off”, in his own words, and he took French courses at a cultural centre. Before joining the ICRC, he had worked for UN agencies, other NGOs and news media. He was recruited as an Arabic-speaking communicator after French was no longer a “big requirement” (Excerpt 4, line 2).

Excerpt 4. Interview with Alex, 22-02-2017.
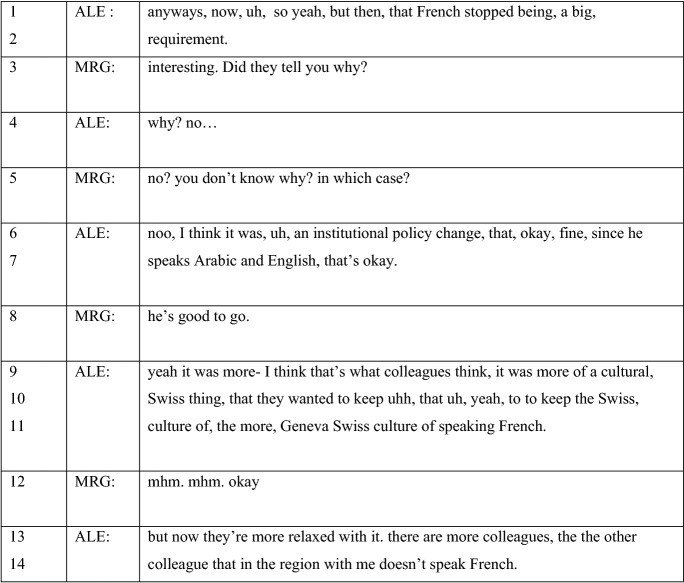


In Excerpt 4 above, Alex links French to “culture” in Francophone Switzerland and Geneva (lines 9–11), in line with authenticity ideologies, and to a specific group of expats: Swiss Francophones who feel more relaxed in French (line 13). He categorises French as not only “from somewhere”, like Paul, but owned by a specific group to which he and his colleague do not belong (lines 13–14), the authentic Swiss Francophone delegates. In Alex’s account, French is presented as a terrain for exclusion and local distinction. This contrasts with the top-down policy change that he describes, which constructs an elite multilingualism centred on the languages spoken in MENA operations, namely English as an international lingua franca (as not all expats speak Arabic) and Arabic as a regional lingua franca for external communications, instead of French as an administrative language orienting to Geneva as a local centre of authority.

For another Arabic-speaking communicator, Adam, French was also a deal breaker for employment at the ICRC despite his previous humanitarian experience. To improve his French, he requested a Francophone mission with a UN agency. Once he became a delegate, Adam did not use it for communications work at the ICRC, where English was the internal lingua franca, as explained above. Nonetheless, his Swiss manager preferred French to better express himself and to have confidential conversations, mainly with other Francophone Swiss, which corroborates Alex’s account above. Adam claimed that French is an “added value” and internal language for the Swiss managers because it “helps to- to maintain the communication channels, you know, help the colleagues to, u:h, express themselves better or just basically, you know, it’s a language to use when you don’t want anyone to understand” (interview, 08-03-2017). Therefore, the need for Arabic speakers in key MENA operations coexisted, not without tensions, with the maintenance of French as an administrative “headquarters” language explicitly required for management posts historically occupied by Swiss delegates and informally used for internal, even confidential, communication among this population.

At a crossroads between authenticity and anonymity, multi-layered elite multilingualism increasingly oriented to new regional centres of linguistic authority besides Francophone Geneva and the Anglo-centric humanitarian sector. There were also fluctuations in the relative value accorded to different working languages in terms of roots or routes.

### Recruitment campaigns for multilingual delegates in a global workforce (2016–2020)

In April 2016, I attended an “international career day” in Lausanne, co-organised by the Swiss Confederation, targeting recent university graduates. The participating institutions included over 50 UN and international organisations. All the materials, presentations and my own conversations were in English, with the marked exceptions of the *Organisation International de la Francophonie*, the Canadian Embassy and the ICRC. This event tapped onto the traditional pool for ICRC delegates: Swiss university graduates in the Francophone region. My conversation with two recruiters at the ICRC stand, in my role as a researcher, confirmed the centrality of French for new candidates (see Excerpt 5 below). Despite my fluency in French, my racialised embodiment and my foreign accent in French might have motivated the recruiters’ choice of English.

Excerpt 5. Fieldnotes from International Career Day, 14-04-2016.

They speak to me in English and I introduce myself and my study on recruitment criteria for the ICRC. The younger one starts telling me what is required to work for the ICRC: […] languages spoken should include English and one of the “languages of interest” but the senior recruiter says that preferably French, because “we have neglected French for a long time”. […] This experienced recruiter tells me that French is very important for mobility because they have delegates who don’t speak French and who cannot be assigned to Francophone missions in Africa. This complicates rotations of international staff. Besides, he categorically claims that “nobody can make a humanitarian career without French because you cannot advance”. When I ask if it is related to the fact that the ICRC has its headquarters in Geneva, he insists that it is not because of that but because of field postings.

The more experienced recruiter defined a “language of interest” as preferably French, which according to him had been neglected institutionally (see Excerpt 5). Unlike delegates in earlier decades (see Excerpts 2 and 4), he did not align with ideologies of authenticity linked to the ICRC’s roots in Geneva. Instead, he focused on the shortage of Francophone delegates to deploy in former French colonies in Africa. This argument was also advanced by the *Médecins sans Frontières* representative at the Fair. The recruiter constructed French as an anonymous language for instrumental communication in a larger region and rejected its framing as an authentic language associated with a local scale. This discursive shift constitutes a scale jump in a multi-layered multilingualism in which working languages such as French and Arabic are linked to a regional scale whereas English is a must-have lingua franca linked to a global scale, as in other international organisations.

During the ICRC presentation at this event, the only one in French with accompanying slides in English, the experienced recruiter explained that it was important to speak English “quite well” and he also pointed out that French and other “languages of interest” were needed, in a descending hierarchical order. This is the case because the ICRC, unlike other organisations, does not have any implementing partners in the field and only collaborates with Red Cross/Red Crescent national societies. According to the recruiter, “languages become important because we are closer to the population” (fieldnotes, my translations from French). The slides strengthened these requirements: “Excellent English and French + other languages an asset”, thus putting the two administrative languages on an equal footing as requirements. In contrast, the UN professional and higher categories require “excellent command of either English or French” and “knowledge of an additional language is an asset but not required for most jobs” (UN careers, 2020). Languages other than English or French are non-compulsory “assets” to obtain these prestigious positions in a hierarchical construction of multilingualism.

**Figure 3:**
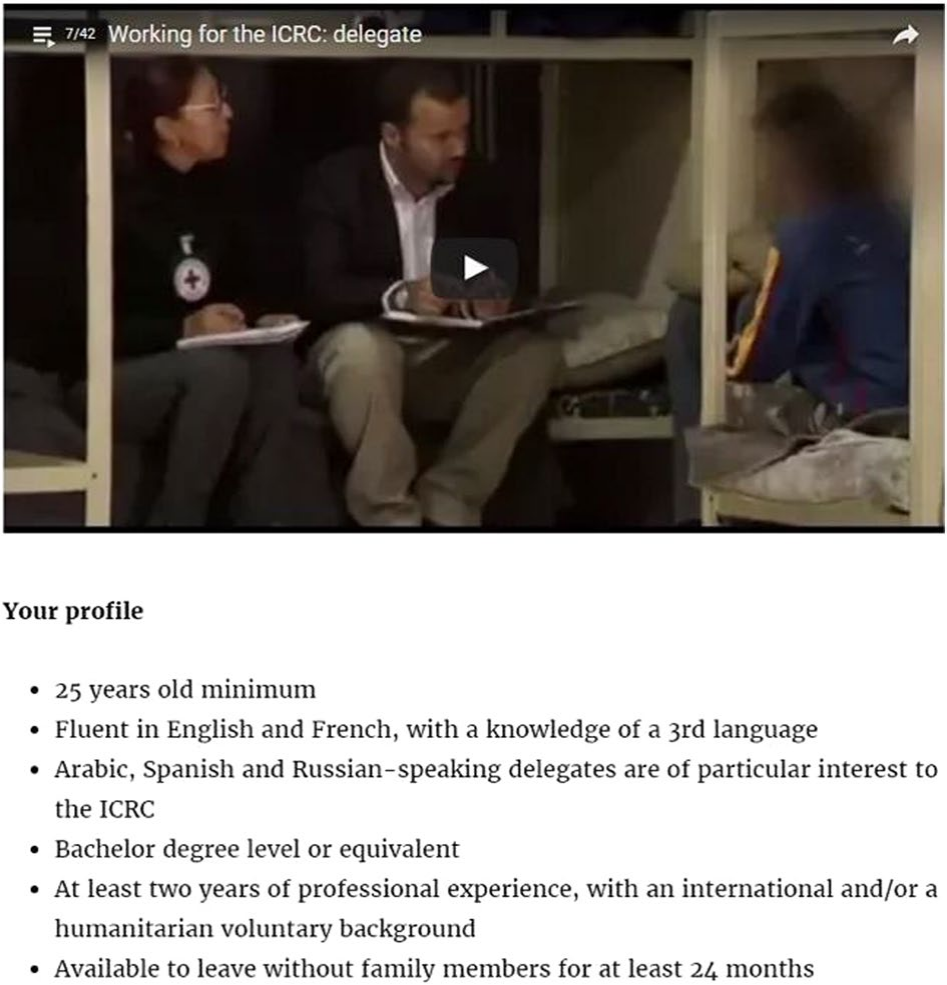
Job advertisement from ICRC website. 17 May 2016 (*Source*: ICRC webpage)

In May 2016, the ICRC advertised new “delegate” positions to work in the field. The job advertisement (see Figure 3 below) requires candidates to be “fluent in English and French with knowledge of a 3rd language”. This further reinforces the institutional importance of French on par with English in contrast to previous campaigns in which only “good command” (2004) and “good grasp” (2012) was expected of delegates. This construction of elite multilingualism strengthened the institutional administrative languages at headquarters and the strategic importance of regional languages for operations.

For the first time in my dataset, delegates were required to speak a third language, which was no longer considered “an asset”. The ICRC prioritised Arabic, which was in high demand for the on-going operations in MENA, alongside Russian and Spanish. However, the ideal profile of a multilingual candidate with international humanitarian experience was hard to find. In practice, recruiters ideally wanted a candidate with “a good English level” as a must-have and two other working languages, but not necessarily French (interview with mobile communications pool manager, 18-03-2016). Out of this campaign, the ICRC could not recruit enough Arabic speakers for on-going operations and as a result, a new recruitment campaign for “Arabic-speaking delegates” was launched in November 2016. It advertised the same profile with different linguistic requirements: “Fluent in Arabic and English, with a working knowledge of either French, Spanish or Russian” (webpage, 03-11-2016). The fluctuating value accorded to French as a working language linked to a world region (West Africa), on par with other working languages such as Spanish, coexists with its institutional role as administrative language linked to Geneva, which was erased in this later campaign. The requirement for fluency in both English and French was abandoned, like in previous campaigns (see Excerpt 4), based on the connection between English and a global scale, as a lingua franca “from nowhere”. These different recruitment campaigns primarily orient to different centres of linguistic authority within the organisation, i.e. MENA operations and Geneva headquarters, with different linguistic regimes and needs.

There are tensions over the importance of French for the new ICRC generations, linked to the issues of career advancement and fluctuating needs in the field, with many key operations in regions where Arabic is lingua franca and an array of Francophone missions in Africa. The compromises on the role of French to recruit suitable profiles, resulting in the use of English as lingua franca, have left many unhappy at headquarters. They mobilise the discourse of “diversity”, rather than origins and tradition, in favour of French. When I first met one of my informants at headquarters, this young Francophone complained the ICRC was becoming “monolingual in English” because more and more employees spoke but English, which he opposed to widespread French–English bilingualism in the 1990s when “the effort of opening up to different languages and cultures” had succeeded (fieldnotes, 20-11-2015).

In 2017, the brochure “Working for the ICRC”—first published in 2012—was re-edited and translated into Arabic, Spanish, Chinese and Portuguese, while the first edition had been only available in English (see previous section). This decision to translate it into “regional languages” of strategic interest might respond to a gradual broadening of the recruitment pools outside Switzerland and Europe, in line with the delocalisation of some services like IT, translation and finance. There were also some crucial HR transformations towards a unified system of posts and salaries for a single, global workforce, which were enforced in 2018 (delegate, personal communication, 2019). Within this unified staffing framework, the 2020 recruitment campaign states that generalist delegates “are fluent in English and two of the following languages: Arabic, Russian, French, Spanish”. This gives more importance to English and demotes the role of French as an administrative language, now likened to other “working languages” (see Excerpt 6). Non-European regional linguas francas (Dari/Pashto or Hausa) are specifically defined as an “asset” for the first time. This timidly broadens the construction of multilingualism based on strategic needs in the field.

Excerpt 6. “Generalist delegate” job description. 12 February 2020 (Source: ICRC webpage)

Fluent command of English and one other ICRC working language: French, Arabic, Russian or Spanish. Knowledge of a third ICRC language or any other language of interest (e.g. Portuguese, Hausa, Dari/Pashto) as an asset.

Excerpt 6 constructs a multi-layered elite multilingualism to access these prestigious posts as expatriate humanitarians. On top we find English as the *sine qua non* for routes linked to a global scale and ideologies of anonymity, followed by “working languages” such as Arabic and French for broader communication in world regions, but which might still be heard as “voices from somewhere” linked to centres of authority like Geneva or Beirut (see Garrido, 2017), and a new bottom layer including languages “of interest” for missions as an “asset”, mainly non-European languages used in on-going humanitarian missions. It remains to be seen if and how non-European languages will be institutionalised as this is a recent development.

The question today is how to diversify international and multilingual profiles against the background of an on-going language ideological debate about the status of English and French and the practical need for Arabic. The incipient delocalisation of services and the staffing system for a unified global workforce, emphasising routes, will be pitted against the symbolic and pragmatic centrality of the Geneva roots in the years to come. Yves Daccord, Director-General of the ICRC until March 2020, has insisted on the centrality of Geneva on several occasions (see above). The nomination of Robert Mardini, of Lebanese origins and settled in Switzerland, as his replacement in April 2020 was welcomed in the Genevese press: “Speaking Arabic, he has an important advantage when we know that 70% of ICRC operations are located in the Muslim world” (Bussard, 2019). Together with the unfolding HR model aiming for one global workforce, this nomination might open a new chapter in the management of languages.

## Betwixt roots and routes: broadening and re-scaling English-centric multilingualism

Based on archival and ethnographic data, this article has articulated major institutional transformations, gradually re-orienting the ICRC from its Geneva roots to expanding humanitarian routes, with on-going language ideological debates over the role of French vis-à-vis English and the resulting language requirements for delegates from 1989 to 2020. Overall, the meaning of “internationalisation” has been contested and re-signified by delegates and managers at headquarters. It has been closely associated with scale shifts from gradual Europeanisation via Swiss-like, Francophone profiles to a practical need for “deswesternisation” (2000s) and a “global workforce” (2016–2020) for humanitarian missions. These scale shifts have broadened elite multilingualism for delegates: besides English as the unquestioned global language, the ICRC has multiplied the required “working languages” on a regional scale and has recently diversified the “languages of interest” to non-European lingua francas as “assets”. Meanwhile, French as the “parent language” has been likened to any other working language for routes, detaching it from the ideological basis of authenticity, but it simultaneously remains an administrative language closely associated with managerial posts and headquarters.

Initially, “internationalisation” was a shorthand for the gradual opening of “delegate” positions to non-Swiss nationals in 1992 in institutional and academic accounts. On an “international” market, English–French bilingualism established a continuity with the earlier Swiss profile. Throughout the 1990s, there were two coexisting centres of authority, Geneva headquarters representing an authentic voice through French, and the global humanitarian field, favouring English as a lingua franca for everybody. At the turn of the century, there were increasing numbers of non-Swiss delegates with a similar “Western” (linguistic) profile. Swiss delegates and managers discursively linked “internationalisation” with discourses of “Anglosaxonisation” opposed to the maintenance of “diversity” at headquarters, indexed by French on a local scale. During the 2000s, there were also calls for “dewesternisation”, i.e. recruiting profiles which were not Swiss-like in terms of linguistic repertoires, education and experiences, in institutional and academic circles. Despite the institutional designation of French as an administrative language equal to English (2003), the relaxation of French requirements coincided with the emergence of Arabic as a sought-after resource for key operations. Elite multilingualism was reduced to English and fluctuating competences in French with a third working (regional) language as an asset.

Recent campaigns mark a multilingual turn embedded in a HR “global workforce” project, seeking to transcend the European/Western scale as an organisation. In 2016, generalist delegates were required to be trilingual in two campaigns orienting to different regional centres of authority, *la Francophonie* and the Arabic-speaking world respectively. Recent recruitment campaigns construct English-centric multilingualism linked to the figure of the delegate, now called “mobile staff”, foregrounding the importance of mobility and routes at the ICRC. French has been likened to any other second “working” language for mobility to certain regions, while new “languages of interest” include non-European lingua francas like Dari or Pashto as a bottom layer. This broadening of multilingualism linked to routes clashes with on-going discourses of English as a threat to “diversity” in Geneva.

“Internationalisation” is understood differently not only at particular times but also by particular social actors that (re)produce different grand narratives identified by Brühwiler et al. (2019). The “traditionalist” narrative among “Swiss delegates with ample field experience” foregrounds the importance of institutional history and Swiss values like consensus for organisational culture (p. 14). They regard “internationalisation” as a negative development towards “feeling like the UN” as in Gerard’s opening quote. In this narrative, French is closely linked to institutional culture and Swiss origins (see Excerpt 2). Even recent non-Swiss recruits like Alex and Adam regarded French as an “authentic language” linked to Geneva and embodied by Swiss Francophone managers, which shows the continued importance of roots and the local/national scale. The traditionalists find the language shift from French towards English as a main lingua franca alienating and indexing a shift in institutional identity (Brühwiler et al. 2019: 14), abandoning authenticity as a basis for linguistic authority benefitting Swiss(-like) candidates. Concerning delegate recruitment, this positioning resulted in the early quotas for non-Swiss candidates in 1990s (Julier, 2002) and the search for “Swiss-like” profiles via French competences. The “internationalist” narrative, favoured by non-Swiss and newer delegates, conceives of the personnel “internationalisation” as necessary and positive (Brühwiler et al. 2019). The dominance of English over French, which translates into accommodating those “international” staff who are “only” fluent in English, seems to be a “natural” development but some are puzzled by this transition in Geneva (Brühwiler et al. 2019: 14). Generally, this has translated into a relaxation of French requirements and a timid re-scaling of elite multilingualism to encompass more non-European languages and speakers as we saw earlier.

Hiring requirements at this humanitarian organisation construct different sets of oppositions orienting to multiple centres of linguistic authority. This paints a more complex, layered and shifting picture of elite multilingualism than in the on-going debates on the institutional role of French vis-à-vis English. For HR, English has been a must-have language since 1989, as an emblem for international humanitarian routes. It contrasts with the “parent” language, French, and other working languages, which are connected with specific regional scales. In turn, HR recruiters have re-scaled French as an authentic index of institutional roots in Geneva to a regional language for mobility in missions, linked to anonymity. Both coexist, as French remains advantageous to work at headquarters unlike other working languages like Arabic (see Excerpt 3). Among the ICRC’s working languages, whose list resembles that of UN official languages, Arabic stands out because of the on-going MENA operations. Although working languages other than English have equal status in the latest recruitment campaign, their fluctuating value linked to operational needs explains the erasure of Chinese in my data. In order to increase the agency’s impact and adaptation to missions, strategic multilingualism has been extended to major regional lingua francas like Hausa and Pashto, thus “dewesternising” elite multilingualism even though they are still “assets” at the bottom layer of this English-centred multilingualism. The tensions between roots and routes, the national and the global, the authentic and the anonymous are also felt in other international(ising) institutions. Given the unique nature of the ICRC, a comparison with multilateral agencies (especially UN) and international NGOs, especially those with headquarters in different regions, would be particularly welcome.

## Data Availability

The institutional documents that I have collected are recruitment materials (e.g. job advertisements, brochures) that are not confidential. The vast majority are available at the ICRC library or were gathered on the ICRC webpage. The only exception is the job advertisement which is partially reproduced in Excerpt 3, which was sent to me by an informant after our interview. Interviews and fieldnotes are not publicly available. They are stored in a confidential way and are not shared with third parties.

## Footnotes

1 Despite this “internationalisation” of personnel, the original Committee of 25 members is still formed by co-opted Swiss nationals, with a revolving door between top government officials and the ICRC presidency (see Forsythe 2005; Brühwiler et al. 2019), with the aim of preserving neutrality and independence in spite of past critiques (see Forsythe 2005; Palmieri 2012).
